# Bioinspired Materials: From Living Systems to New Concepts in Materials Chemistry

**DOI:** 10.3390/ma12132117

**Published:** 2019-07-01

**Authors:** Corinna F. Böhm, Joe Harris, Philipp I. Schodder, Stephan E. Wolf

**Affiliations:** 1 Department of Materials Science and Engineering (WW), Institute of Glass and Ceramics (WW3), Friedrich-Alexander University Erlangen-Nuremberg (FAU), Martensstrasse 5, D-91058 Erlangen, Germany; 2 Interdisciplinary Center for Functional Particle Systems (FPS), Friedrich-Alexander University Erlangen-Nuremberg, 91058 Erlangen, Germany

**Keywords:** biomimetic materials, emergence, biominerals, non-classical crystallization

## Abstract

Nature successfully employs inorganic solid-state materials (i.e., biominerals) and hierarchical composites as sensing elements, weapons, tools, and shelters. Optimized over hundreds of millions of years under evolutionary pressure, these materials are exceptionally well adapted to the specifications of the functions that they perform. As such, they serve today as an extensive library of engineering solutions. Key to their design is the interplay between components across length scales. This hierarchical design—a hallmark of biogenic materials—creates emergent functionality not present in the individual constituents and, moreover, confers a distinctly increased functional density, i.e., less material is needed to provide the same performance. The latter aspect is of special importance today, as climate change drives the need for the sustainable and energy-efficient production of materials. Made from mundane materials, these bioceramics act as blueprints for new concepts in the synthesis and morphosynthesis of multifunctional hierarchical materials under mild conditions. In this review, which also may serve as an introductory guide for those entering this field, we demonstrate how the pursuit of studying biomineralization transforms and enlarges our view on solid-state material design and synthesis, and how bioinspiration may allow us to overcome both conceptual and technical boundaries.

## 1. Introduction

Human society is confronted with an ever-increasing demand for energy, high-performance functional materials, and cheap and lightweight structural materials. Today, we are forced to rethink the way we deal with earth and nature’s resources. For decades or even centuries, we have overexploited the limited resilience of global and local eco- and climate systems. Archaeological findings show that human misconduct has repeatedly led to the local decline of advanced human civilizations. Now we are facing comparable issues on a global scale. There is thus a pressing need for new material concepts, which lead to resource-friendly and environmentally compatible materials. New materials should have extended lifetimes due to resilience, exhibit re- and up-cyclability, and be energy efficient, e.g., due to lightweight designs. Simply put, new materials must be eco-friendly throughout their entire lifecycle, but must reach our demands for their functionality, performance, and endurance. In this venture, biogenic functional materials may guide us to a systematic understanding of materials, with a new focus on how a material impacts an (eco)system, its consumption of resources during manufacture and usage, and the afterlife of its remnants. 

Evolution optimizes the performance of biomaterials as this increases the odds for survival. A second and often overlooked quality of evolutionary optimization is the amount of energy which a creature has to invest to generate a certain material/function. Thus, where possible, abundant and degradable materials such as calcium carbonate or silica are employed in order to generate mineralized tissues, and remarkably mild synthesis processes have been developed. Moreover, beyond mere functional performance, biogenic materials are also optimized in regards to their functional density (e.g., by functional gradients). The resulting material is formed from an energetically “cheap” synthesis from abundant resources, and has maximized endurance before catastrophic failure [1,2,3,4]. For this to be achieved, biogenic materials have to exhibit a combination of strength and toughness, properties that are key in the design of man-made materials [5].

In this review, we focus on bio-inorganic solid-state materials, i.e., biominerals, which fulfil several mechanical functions ranging from structural support, to protection, motion, and sensing [1]. Through representative examples, we will demonstrate how the detailed analysis of biomineralization processes in organisms has affected the view on materials design and materials synthesis.

## 2. From Creatures to Concepts

### 2.1. From Calcareous Creatures to New Concepts in Crystallization

**Figure 1:**
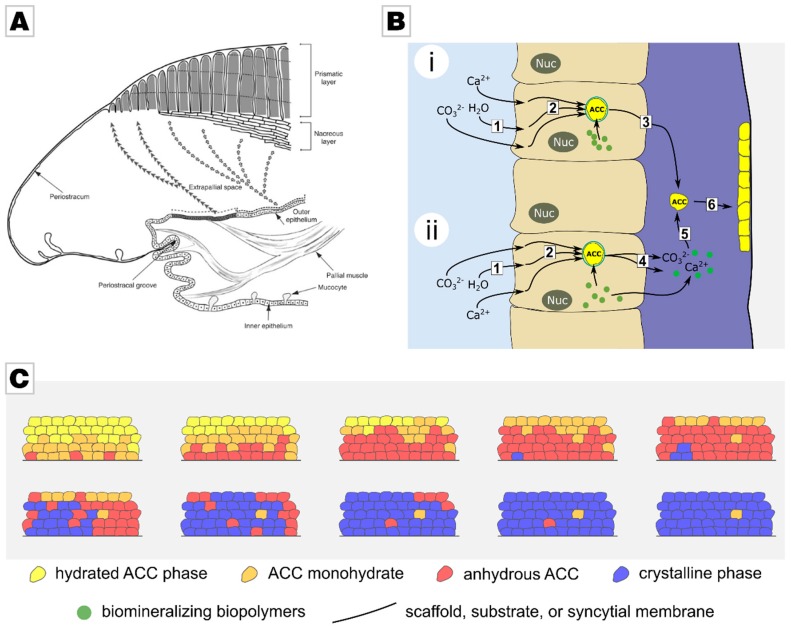
(**A**) Shell mineralization occurs in two compartments, the mantle and the extrapallial space. Reprinted from Marin et al., ref. [6], with permission from Elsevier. (**B**) Two generic mineralization pathways possible within the second compartment. (1) Uptake of ions from the environment. (2) Ion storage in intracellular compartments, e.g., as amorphous calcium carbonate (ACC) nanoparticles. (3) Exocytosis of calcium carbonate can occur by redissolution to ions or by the export of the stored ACC granule. (5) ACC colloids can reform from ions, under process-guidance of biopolymers. (6) Attachment of nanoparticles to the growth site. Reprinted from Wolf et al., ref. [19], with permission from Elsevier. (**C**) Transformation of hydrated ACC to crystalline calcite; starting with highly hydrated ACC, losing water, and by first forming monohydrate and then ACC, before crystallization. Reprinted from Wolf et al., ref. [19], with permission from Elsevier.

Biomineralization processes are highly complex and exceptionally well controlled. In molluscs such as bivalves and snails, shell formation is conceptually simple when compared to other biominerals such as bone. This process is analogous to continuous manufacture and starts with the transformation of a larva to a juvenile shell [6]. During this process, a thin membrane is formed from chitin, called the periostracum. This membrane separates the shell mineralization process from the (marine) environment and serves as a substrate on which the mineral is deposited (Figure 1A). The process of calcification takes place in the extrapallial space, a liquid-filled and narrow space between the outer mantle cells, which drive the mineralization, and the periostracum [7]. The inner mantle cells are in contact with sea water and are responsible for the uptake of the ions required for calcification from sea water. These ions are then directed towards the outer mantle cells and are stored there in the form of amorphous calcium carbonate (ACC) granules within vesicles [6]. In the extrapallial space, the shell is generated by self-organization. This process is temporally and spatially controlled by the mantle cells via secretion [8]. The liquid in the extrapallial space is rich in proteins, glycoproteins, acidic polysaccharides, chitin, and lipids; it bears all the inorganic, organic, and regulatory components that are needed to generate the mollusc’s shell. The organic components are thought to interact with the forming mineral in two ways: They inhibit unwanted precipitation and simultaneously direct mineralization to the desired site [6,9]. Often these biomineralization-active biopolymers are remarkably acidic [6,10,11,12,13], much more acidic than common proteins. The exact process which controls shell formation is still disputed; but recent works on nacre formation have demonstrated that the mineralization is mainly driven by nanoparticle attachment rather than by an ion-wise driven growth [14], as suggested by classic textbook theories. This route which proceeds via nanoparticle assembly probably increases the growth rate, as crystal growth is in the range of several tens to hundreds of picometers per second [15,16]. Further, it allows the mollusc to spare water and thus to address a logistical issue of transporting a sufficient amount of scarcely soluble mineral to the mineralization site without removing large volumes of water from this site [17,18].

Various generalized pathways of biomineralization have been proposed (Figure 1B) [19,20]. One possible pathway is that cells secrete stored mineral granules, which then attach to the site of growth. According to an alternative pathway, the stored mineral granules are re-dissolved, and ions are released. Mineral nanoparticles then reform, under the guidance and process-directing action of biopolymers present in the extrapallial fluid [21,22]. In both cases, mineral granules attach to the mineralization site [19]. After the attachment of the nanoparticles, the transformation to a crystalline state takes place [23,24]. Organic molecules, which initially inhibit and then guide mineralization, are entrapped into the mineral during mineralization, as they adsorb onto the attaching nanoparticles [6,9,25]. The matrix-mineral interaction of these organic molecules is ascribed to several physicochemical functions, such as polymorph selectivity [26]. These interactions are thought to give rise to the hierarchical structure of the monolith by controlling crystal growth rates along selected crystallographic axes [6]. Crystallinity of the forming biomineral travels through the amorphous body following a random path by homoepitaxial nucleation. For this, the initially highly hydrated mineral precursor has to transform into an anhydrous state before crystallization can occur [27,28,29,30,31,32] (Figure 1C). As crystallization proceeds granule by granule, a granular structure at the nanoscale is preserved in the final biomineral, strongly affecting its properties [29], a fundamental feature that is discussed later.

In summary, biominerals often form via space-filling accretion of colloids [19,23,33,34], which consist of, or form from, a transient precursor phase. The formed glassy body then transforms into a mesocrystalline mineral body [23,27,35,36,37,38]. Depending on its degree of mosaicity, the biomineral can behave like a single crystal although it is composed of individual crystalline nanograins and thus resembles a hybrid ceramic rather than a crystal. This generalized view of biomineral formation, that it takes place via attachment of precursor nanoparticles, is in conflict with classical crystallization theories. In the well-established and often-tested framework of classical crystallization, only ions and single molecules are considered as fundamental building blocks of the final crystal [29].

In the last decades, an overwhelming number of reports on bio-inspired in vitro crystallization experiments have been published and have revealed that this apparent conflict not only arises from a “vital effect”, thus from the involvement of cells, but that non-classical processes can be generated in vitro simply by adding polymeric species, such as block-copolymers and polypeptides. These “process-directing” species essentially stabilize initial stages of mineralization, e.g., by acting as capping agents. In accordance with the LaMer model, sufficiently high supersaturation can cause a high particle number density generated by burst nucleation. If these initial stages of mineralization are caught and stabilized, the resulting solids interact according to colloid chemistry. The final outcome—if particle interaction potentials come into play which control their self-assembly—is the genesis of a crystalline body via accretion of entities, which are far larger than single ions/monomers.

Cölfen, Antonietti, De Yoreo, and many others have clearly exemplified this for the process of oriented attachment, in which nanocrystals are generated, which are then face-selectively stabilized, often by block-copolymers but also by other ionic solute species, e.g., ions. Accretion of the nanoparticles is then driven by a decrease in surface energy, when the non-polymer-functionalized nanocrystal surfaces crystallographically align and come into contact. Li, De Yoreo, and co-workers demonstrated, by high-resolution transmission electron microscopy using a fluid cell, that iron oxyhydroxide nanoparticles undergo oriented attachment after various interparticle configurations are screened by continuous rotation and interaction before, upon perfect lattice match, a sudden jump to contact takes place [39]. This crystal growth mode can generate exceptional anisotropic and high-surface-area morphologies [40,41,42,43]. 

A distinctly different non-classical crystallization process was first observed in 1997 by Gower & Tirrell [44]. This process is induced by nucleation-inhibiting polymers—mimicking the action of poly-aspartate rich biomineralization polymers—which trigger/allow for liquid-liquid phase separation of the mineral. The liquid-like and thus amorphous mineral precursor becomes the active agent of mineralization. The transient liquid can be transformed into a range of morphologies, e.g., by infiltration or coating. Upon dehydration, these non-equilibrium morphologies then transform into mesocrystalline mineral bodies while retaining their initial morphology [44]; a process which is called pseudomorphic. This assembly of mineral precursor nanoparticles, dubbed the polymer-induced liquid precursor (PILP) process [44], shows remarkable similarities to biomineralization processes of calcifying organisms, both in mechanistic and structural aspects [22,45]. It was often questioned whether this process is simply based on coacervation but it was demonstrated that a liquid-condensed and thus highly hydrated calcium carbonate precursor phase can form even in the absence of polymers [46].

Both non-classical pathways, via oriented attachment and via liquid-condensed precursors, allow for control of the nanoscale organization of the final mineral. The formidable mechanical characteristics of biominerals demonstrate this clearly. By strict control on the nanoscale, material properties can be dramatically enhanced. In fact, all material properties which originate or are, at least, partially active on the mesoscale, can be affected by non-classical, particle-mediated crystallization pathways. Today, non-classical crystallization processes are consequently of high interest due to the possibilities of tailoring mechanical, magnetic, optical, phononic, thermoelectric, or photonic properties or the surface area of materials [47,48,49,50,51,52,53,54,55,56,57,58,59,60,61,62]. This highly active field, which can be seen as a new field of solid-state materials chemistry, profits and is motivated by the detailed analysis of biomineralization and biomimetic crystallization studies by revealing the short-comings of the classical crystallization models [19,29,63,64,65,66,67,68,69].

### 2.2. Biominerals as an Evolutionarily-Tested Archive of Functional Material Design Motifs

The minerals which comprise biominerals, e.g., calcite and aragonite or hydroxyapatite, are mostly brittle in their pure geological form [70,71,72,73,74,75]. In biominerals, these minerals simultaneously feature high strength and high toughness [70,76,77,78]. The origin of this phenomenon is the blending of the mineral phase with a minor fraction of organic matrices ranging over numerous length scales to generate hybrid and composite materials. The underlying design concepts are of high interest for the development of modern materials but require a good understanding of the hierarchical organization of these bioceramics, their macro- to micro- to nanoscale structures and the strengthening and toughening mechanisms that emerge from this complex structural organization.

**Figure 2:**
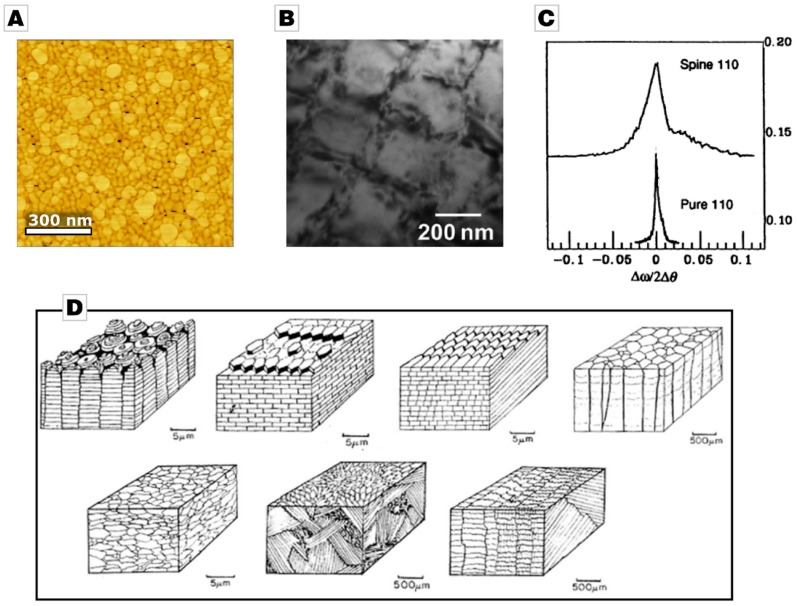
**Figure2.** Structural organization of biominerals in bivalves. (**A**) Atomic force microscopy (AFM) phase image of *Glycymeris glycymeris* treated with EDTA. Reproduced from ref. [81], reproduced with permission. (**B**) TEM image of *Pinctada fucata.* Reprinted with permission from ref. [92]; copyright (2012) American Chemical Society. (**C**) XRD diffractogram of a macroscopically single-crystalline sea urchin spine and pure calcite demonstrating Debye-Scherrer broadening of the biocrystal. Reprinted from ref. [95]; reprinted with permission from AAAS. (**D**) Seven different microstructures in bivalves and gastropods: Columnar nacre, sheet nacre, foliated, prismatic, crossed-lamellar, complex crossed-lamellar, and homogenous. Reprinted from ref. [103] with permission from John Wiley and Sons.

A reoccurring motif is the nanogranular structures that have been observed among a wide range of biominerals of different species and phyla, i.e., human bone [79] and kidney stones [80], shells of bivalves [81,82], cephalopods [83], gastropods [84], sponges [72], egg shells [85,86], sea urchin spines [87], and sea urchin teeth [31], see also ref. [19] and references therein. Typically, this nanostructural feature has been identified by atomic force microscopy (AFM) phase imaging in combination with etching treatments [83,84,88,89,90,91], but transmission electron microscopy (TEM) analysis [91,92,93] and X-ray diffraction (XRD) analysis have also corroborated these nanogranular structures [94,95] (Figure 2A–C). Additionally, solid-state NMR is an invaluable, and non-destructive method for the analysis of the organic-inorganic interface. It has commonly been used to show that an intracrystalline amorphous fraction of the inorganic matrix is co-localized with the intergranular organics [96,97,98,99]. Nanogranularity seems to be a common feature across species, even in those that are only distantly related and composed of different minerals, e.g., calcium phosphate, calcium carbonate, calcium oxalate, or silica. Beyond this unifying feature on the mesoscale, the structural organization on the microscale deviates remarkably between species and biominerals as they are adapted to their function and requirements [100,101]. Combining different microstructures in different parts of the mineralized tissue allows biomineralizing organisms to form functional gradient materials with (probably) only minor changes to the mineralizing machinery [102].

Taking molluscan shells again as an example, seven different calcareous microstructures are known: Foliated, prismatic, crossed-lamellar, complex crossed-lamellar, homogenous microstructures, and, additionally, two different nacreous structures [103] (Figure 2D). Nacreous, crossed-lamellar and complex crossed-lamellar structures are typically composed of aragonite whereas foliated, prismatic, and homogenous structures are mostly made from calcite [104]. The mechanical properties of these microstructures are distinct due to the different minerals used and the diverse structures into which they are formed. Each microstructure is thought to offer different mechanical advantages. The most widely investigated microstructure is nacre, which exhibits the highest tensile and compressive strength, whereas the crossed-lamellar structures, e.g., in the Queen Conch *Strombus gigas*, exhibit the highest fracture toughness [70]. Notably, interest in the apparently simple prismatic microstructure has also increased over recent years [82,105,106,107,108,109]. Whereas the crossed-lamellar layer is often the sole microstructure present in a shell (for instance several crossed-lamellar layers of different orientations are present in both *Strombus gigas* and *Glycymeris glycymeris* [81,110,111]), nacreous layers are often found in conjunction with prismatic layers. 

There are some hidden but common design principles that these microstructures share. The structural elements of which these microstructures are composed feature some re-occurring characteristics: First, they typically have an extreme aspect ratio and/or a high surface/volume ratio—be it lamellae in cross-lamellar structures, tablets in nacre, or concentric layers in osteons or glass sponge spicules. Second, the characteristic length of the short axis is in the nanometer range, e.g., a few tens to hundreds of nanometers in the case of lamellae and nacre tablets. Third, the elements pack densely and the interelement volume is composed or, at least, enriched with an organic matrix. Remarkably, the smallest building units, i.e., the nanogranules, also perfectly comply with this set of rules. 

The stomatopod dactyl club is an impressive example of a highly damage-tolerant smashing tool, which is perfectly adapted to prevent catastrophic failure. This is achieved by the design of the microstructure, which comprises a highly mineralized helicoidal chitin layer, which minimizes internal damage of the repeatedly highly loaded club [112]. It goes without saying that this exceptional example has already led to a number of mimetic attempts demonstrating the efficacy of this approach [113,114,115].

The core design concept in these examples is deliberate generation and maximization of weak interfaces, which, upon load and failure, then dominate the fracture mechanics. The large aspect ratio of the building units leads to crack deviation, tortuous, and massively enlarged crack paths, and pull-out effects. The organic layer, which is typically located at the interelement interfaces, increases the dissipation of the crack energy due to its toughness and can also lead to enhanced stress delocalization, auxetic behaviour, or even self- or crack-healing capabilities. What we observe is a formidable example of failure design, the optimization of an unwanted but inevitable material response to external and potentially deadly threats. This beautifully demonstrates that evolution not only promotes performance but also the endurance of a biomaterial, as the failure or fatigue of a weapon, armature, tool, or sensor is equivalent to the death of the individual organism.

The adaption of this approach for new materials thus requires deliberate introduction of weak interfaces and their functionalization by a suitable organic matrix. Barthelat et al. followed Nature’s guide and demonstrated that, by implementing these structural motifs into a glass body, the brittleness of glass could be overcome and that its toughness could be increased by 200-fold [116].

**Figure 3:**
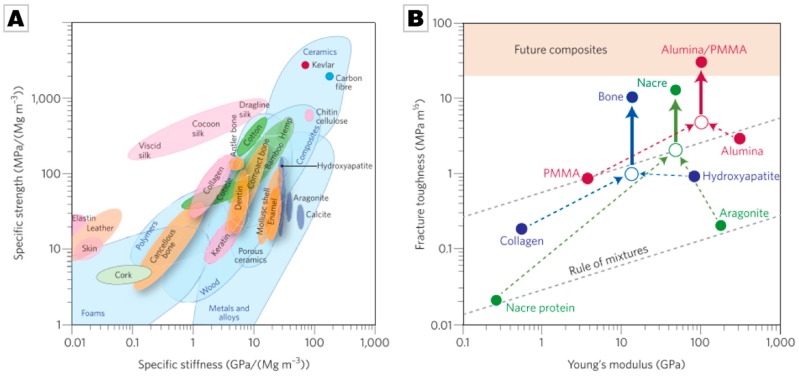
(**A**) Ashby plot, i.e., chart of material properties normalized by density, of natural and synthetic materials. (**B**) Projections for natural and synthetic materials, demonstrating the increase in toughness compared to homogeneous mixtures of their components, i.e., according to the rule of mixtures. By utilizing extensive extrinsic toughening mechanisms, hierarchical materials increase their toughness both for crack growth (closed symbols above the solid arrows) and, albeit to a lesser extent, for crack initiation (open symbols). Adapting these design concepts may lead to synthetic ceramic materials with outstanding properties. For more details, see Wegst et al. [5]. Reprinted by permission from Springer Nature: Nature, Nature Materials, ref. [5], Copyright 2014.

The remarkable toughening and strengthening effects caused by this hierarchical organization and the hybrid and composite layout of biominerals can best be demonstrated by Ashby plots [5,77], see Figure 3. Continuing with the molluscan example, it becomes clear that shells are five times stronger than pure calcite and aragonite, although the density essentially remains constant. This demonstrates that biominerals have overcome the “conflict between strength and toughness” of man-made materials [117].

A range of strengthening mechanisms are known for crystalline materials; in which it is essential to hinder dislocation motion of slip systems as this gives rise to plastic deformation. By “pinning down” dislocations the shear stress required to activate their motion is increased [118]. It should be mentioned that strengthening mechanisms are typically best established for metals but, given that minerals such as calcite exhibit slip-systems, a transfer of knowledge is generally accepted [119]. A range of hardening techniques are known that reduce dislocation motion: Work hardening, grain boundary strengthening, solid solution hardening, dispersion hardening, and transformation hardening. In work hardening, dislocations are introduced into the most compliant slip planes. Grain boundary strengthening, i.e., grain refinement, generates more grain boundaries, which impede dislocation motion by dislocation pile-up at grain boundaries; the increased shear stress required for further dislocation motion is described by the Hall-Petch relationship [120]. Solid solution strengthening causes local lattice distortions and thereby also interferes with dislocation motion. In dispersion hardening, either by particles or by precipitates, dislocation motion is also hindered as dislocations have to either cut through (in case of small, coherent precipitates) or climb the precipitates (in the case of bigger, coherent precipitates, incoherent precipitates, or particles). Cutting as well as climbing requires increased shear stress and thus causes strengthening. In Cu-saturated Al_2_Cu alloys, small, coherent precipitates form upon cooling, which are called Guiner-Preston-precipitates [118].

The failure criterion in ceramics is mostly their low fracture toughness. Thus in (bio)ceramics it is toughening, i.e., hindering fracture, that is of special interest [121]. Toughening can be achieved by several mechanisms, i.e., grain size reduction, crack tip deflection, or crack tip shielding [121]. In grain size reduction, the crack has to intersect more grains with different crystallographic orientation and grain boundaries of different orientation, increasing the force required for crack propagation. Crack deflection can be caused by introduced planes of weakness. Planes of weakness can appear in form of grain boundaries, phase boundaries, fibrous, or plate-like microstructures, inclusion boundaries, pre-micro-cracking, or pre-stressing. Grain, phase, and inclusion boundaries as well as microstructures allow crack deflection. In micro-cracking, pre-micro-cracks are introduced into the ceramic material. As the crack propagates the stress in front of the main crack links the main crack and the micro-cracks. The crack energy of the main crack is thereby reduced. Pre-stressing is done in materials sensitive to tensile stress, such as glasses [122] and cement [123] by introducing compressive stresses into the surface to increase its toughness. The concept of crack shielding requires a process zone, surrounding the crack, applying compressive forces to the crack surface. The process zone might have different origins, e.g., fibres that traverse the crack tip, a plastic zone surrounding the crack tip, or phase transformation of particles. Fibre toughening is mainly caused by delamination and pull-out of the fibres from the matrix. The plastic zone imparts a compressive force onto the crack surface thereby shielding the crack. In all of these cases, crack energy is absorbed increasing toughness [121]. The remarkable increase in strength and toughness of mollusc shells and other biominerals emerges as a wide range of these mechanisms are simultaneously active in bioceramics on different length scales [76,81,124,125,126,127,128,129]. For instance, at the microscale the layered arrangement and the individual microstructural features of shells can be regarded as grain size refinement and thus cause strengthening and toughening [81,110,111,130]. Nacre’s brick-and-mortar structure has been observed to exhibit several strengthening and toughening mechanisms at the nanoscale. Crack deflection around the individual aragonite tablets is the oldest toughening mechanism known in nacre. At the nanoscale several strengthening features have been observed, such as the nanoscale waviness at the surface of the nacre tablets causing strain hardening during deformation [124]. Mineral bridges [131] as well as nano-asperities are observed to increase friction during sliding [132]. Furthermore, unfolding inter-tabular proteins have been observed to bridge cracks and thus increase toughness [133]. The nacreous aragonite tablets themselves have been reported to show increased strength compared to the mineral aragonite [134]. Their nanogranular origin can clearly be correlated to grain size refinement, causing dislocation pile-up and crack deflection and thus certainly show an effect in strengthening and in toughening [19]. Indeed, for the case of hen eggshells, a functional material gradient was identified based on the Hall-Petch-relationship [85]. In addition to the hierarchical arrangement of the mineral, intracrystalline organics have been observed within nacre tablets [133,135,136]. The impact of intracrystalline organic macromolecules on the mechanical properties of the aragonite tablets is still under debate. As the organic macromolecules possess a lower modulus, the crack path should run into the region enriched with organics. Organic macromolecules thus absorb crack energy and thereby toughen nacre tablets [137]. Furthermore, the incorporated organic molecules are thought to significantly impact the strength of the material. They are assumed to act as obstacles in dislocation motion, causing dislocation cuts or climbs, depending on their size and coherence and thereby strengthening biogenic aragonite [109]. Similarly, single amino acids have been observed to cause significant lattice distortions and thereby result in a tremendous increase in hardness and strength [138]. Finally, Guiner-Preston-like coherent precipitates were recently identified in biominerals and biomimetic materials, the presence of which was shown to remarkably impact the mechanical properties of the materials [71].

This preceding list shows that the remarkable properties of bioceramics rest on a range of strengthening and toughening mechanisms. Many of the strengthening mechanisms are comparable to strengthening mechanisms which were first demonstrated in metals, and all the mechanisms root in hindered dislocation motion. Several of the listed toughening mechanisms are known from and are already applied in ceramic materials. Importantly, some of the other mechanisms are newly identified and can possibly be exploited when designing new materials. One may thus raise the question that if all of these motifs were already known, does the field of biomimetics or the analysis of biogenic materials yields new or beneficial knowledge? The key feature from which biogenic materials derive their exceptional properties is their structuration over a number of length scales, giving structured, hierarchical materials. Adapting this fundamental approach to the design of novel materials allows for a further increase in the performance of materials beyond their current limit, see Figure 3B.

Recent reports beautifully demonstrate how efficient this concept is, and future work will entail pushing this approach further to generate materials that exceed current limits. Koyama et al. showed that by mimicking the hierarchical and laminated organization of bone in metastability assisted multiphase steels both crack and fatigue resistance could be increased exceptionally [139]. Ritchie and co-workers demonstrated that the superb damage-tolerance of hierarchical steels that are composed of nano-band austenite and nano-lamellar martensite, a design inspired by the byssus-threads of mussels, crucially rests on the synergistic cooperation of various toughening and strengthening mechanisms [140]. As stated above, weak interfaces often play an important role in strengthening mechanisms such as crack deflection. When transferring such concepts to new material classes, the adhesion/wetting of interfaces must be well attuned. Park, Ritchie, and co-workers recently showed that alumina structures can be infiltrated with a wetting zirconium-based bulk-metallic glass mortar to yield a material with high flexural strength and fracture toughness [141]. Bill and co-workers showed in a study in which a multilayer ZnO ceramic was strengthened with interfacially bound peptides that the fracture behaviour of the layered material can be adjusted by altering the binding strength of the peptide [142]. By selecting a peptide with weaker binding a weaker interlamellar interface is generated, which can be of advantage [142].

As we have focused in this section on the mechanical properties of selected biominerals, it is worth noting that biominerals are not solely optimized for mechanical performance. Instead, evolutionary optimization tunes all aspects of the biomineral that affect the survivability rates of their host organism. This multifunctionality, which elegantly “blurs the distinction between material and device” [143], is rendered possible mainly by the hierarchical organization of the biomineral. If the biomineral serves as (a part of) a sensor, then its materials properties are attuned to this specific function, for instance in brittlestars in which calcite serves in a photoreceptor system [144]. Another nice example is hen egg shells whose primary task is protection of its content but which also serve as a depot of inorganic nutrients, foremost calcium ions. During incubation, the hen eggshell partially dissolves to feed the growing chicken, whilst still preserving sufficient mechanical stability to maintain adequate protection [145]. At later stages of incubation, these redissolution processes change the nanostructure of the eggshell purposefully weakening the shell to ease chick hatching [85].

### 2.3. Biogenic Silica Formation Demonstrates How to Mildly Drive Metal Oxide Formation

A range of species generate silica under exceptionally mild and biocompatible conditions, for instance protists, sponges, diatoms, and various plants. The synthesis conditions of silica skeletal spicules in marine sponges for instance, are in stark contrast to the conditions under which silica can be generated in vitro. Classical synthesis involves elevated temperature/pressure or strong acids/bases. Biosilica is on par with biominerals with regards to its delicate, hybrid, and hierarchical organization [146,147], from which it obtains similar beneficial properties to those observed in crystalline biominerals [148,149,150,151,152,153]. The ability of these lower organisms to synthesize glass fibres with exceptional optical and mechanical properties, which exceed those of man-made optical fibres [146,147,154], naturally invoked immense interest. It was soon realized that some of the organic components, incorporated into the bioglass matrix, were capable of facilitating silica synthesis under bio-relevantly mild conditions [150,155,156]. The silica spicules contain a proteinaceous axial filament composed of silicatein whose primary sequence bears a remarkable resemblance to cathepsin L, a peptide-cleaving protease, which belongs to the papain-like cysteine protease superfamily [153,156]. The protease-derived silicatein showed hydrolytic activity against tetraethoxysilane (TEOS) [156]. Intense research, including the generation of recombinant silicatein along with biomimetic derivatives, finally revealed that the catalytically active centre acts in a similar fashion to cathepsin L in peptide cleavage [156,157]. The details of the catalytic reaction cycle are discussed in detail in a recent review by Morse and Shimizu and are not reviewed here [158,159]. Due to evolutionary relaxation, i.e., the need for other substrates to be hydrolyzed by silicatein [158], silicatein is able to process a wide range of substrates beyond silicon-related or peptide-related substrates even though the superfamily to which it belongs is known for its high substrate specificity. It was shown that (recombinant) silicatein is capable of precipitating various transition metal oxides from different solute precursors, e.g., gallium oxide from gallium nitrate [160] or anatase from a soluble lactato-titanium complex [161]. Silicatein is capable of processing not only metal-centred substrates, but also catalyzing polymerization to give biodegradable poly(L)-lactide [162], silicones [163], or gas-sensing pincer metal complexes [164]. Tremel and co-workers pushed this idea further, by binding silicatein on surfaces by various techniques to give functionalized metals, e.g., gold-thiolate surfaces by means of a nitrilotriacetic acid (NTA)-linker system, and metal oxide-surfaces by an NTA-bearing capping agent [165,166,167,168]. Natalio et al. applied silicatein for the synthesis of hydroxyapatite [169] and calcium carbonate [170]; in the latter case they demonstrated that the forming mineral spicules, self-assembled from aligned calcite nanocrystals, feature remarkably enhanced bending strength.

Various approaches further show that enzymatic activity can be transferred to other non-proteinaceous systems by taking inspiration from the stereochemistry of the active centre of silicatein. By reducing the action of silicatein to its absolutely fundamental principles, it was possible to design catalytically active molecules of lower structural and stereochemical complexity. This corroborated the proposed mode of action of silicatein, but also opened up the possibility of developing inexpensive, robust catalysts for solid-state materials synthesis suitable for chemically versatile scale-up synthesis. The first such example is a group of diblock copolypeptides, with the ability to self-assemble, which showed catalytic activity in the desired neutral range [171]. These studies showed that it is key to combine serine or cysteines as nucleophilic moieties with amine-terminated moieties, such as lysine, to generate similar catalytic activity as in silicatein [153,156,157]. With this lesson learned, Adamson et al. pushed this concept further to a non-peptide system based on the diblock copolymer poly(2-vinylpyridine-b-1,2-butadiene) [172]. However, the rate of silica formation in a benchmarking setup was only two-thirds of the diblock copolypeptides; this was attributed to a change in pK-values of the nitrogen-bearing moieties [173]. Kisailus et al. transferred these concepts to thiolated self-assembled monolayers on gold, by functionalizing one population of gold nanoparticles with hydroxyl-terminated and the other one with nitrogen-terminated thiols. The mixture of the two differently functionalized gold nanoparticles showed catalytic activity, which can be explained when considering the contact zone of the two differently functionalized gold nanoparticles. When in close contact, they build a catalytically active centre, which triggers condensation, eventually entrapping the gold nanoparticles in a silica network. If exposed to the monomer, the individual population of functionalized nanoparticles were inactive; and only showed catalytic activity when mixed together [174].

Finally, small molecules were screened to assess whether they were also capable of mimicking, silicatein activity by stochastic assembly. A range of small molecules bearing two moieties, one nucleophilic group (e.g., –OH, –SH) and a hydrogen-bond acceptor (e.g., a primary or tertiary amine) were screened by Roth et al. [175]. Monofunctional small molecules were used as a control group, which showed barely any activity for silica formation from TEOS. From the set of bifunctional molecules, two candidates were noticeably active, i.e., cysteamine and ethanolamine. Tertiary amines, as expected, showed reduced activity; likewise, the catalytic rates of thiol-bearing molecules excelled those bearing hydroxyl-moieties [175]. 

These findings sparked remarkable interest as the mild synthesis conditions open up a new opportunity to incorporate sensitive and delicate organic molecules into a glass matrix; as a proof of feasibility, Roth et al. incorporated blue fluorescent protein and living *Escherichia coli* into silica [176]. By combining low molecular weight silicatein-analogues with morphogenetic agents, Corma et al. were able to generate meso- and micro-structured silica [177] otherwise only accessible by use of harsh conditions.

From these developments, it becomes clear that research into bio-silification demonstrates the potential offered by enzymatic-based synthesis for solid-state materials, and provides similar opportunities for advanced material design as given by particle-driven mineralization processes.

## 3. Outlook

In this review, we aimed to demonstrate how detailed analysis of biomineralization processes has changed our view on the synthesis and structural design of solid-state materials. We have demonstrated that studies on biomineralization have triggered—or at least catalyzed—a shift towards non-classical views on crystallization. Further we have shown that biominerals follow design rules, which are not only focused on performance but also on characteristics that are key for the design of sustainable materials such as resilience. Furthermore, we reviewed how the study of biosilification has opened new pathways to the synthesis of functional materials under mild conditions. By shifting from a classic solid-state materials route, characterized by harsh conditions, to a biocompatible hydrolytic process, which bears strong similarities to sol-gel chemistry, but under mild conditions. These selected examples, which represent only some aspects of bioinspired materials chemistry, demonstrate that the pursuit of studying biomineralization eventually has the potential to fundamentally transform our view on materials, providing blueprints of how to overcome both conceptual and technical boundaries. As biogenic materials naturally rely on abundant and eco-friendly and sustainable components, the detailed analysis of their concepts and synthesis procedures may help us to overcome some of the critical issues the human society currently faces. Biominerals demonstrate how mundane and ordinary components such as calcium carbonate or phosphate can be transformed into load-bearing, damage-tolerant materials. They show us energy-efficient synthesis routes to a range of inorganic solid-state materials, which are hierarchically structured. Furthermore, biomineralization gives access to hybrid nanoceramics, functionalized and toughened/strengthened with intracrystalline organic matrices, a class of material, which is inaccessible by the classic ceramic process.

## References

[1] Mann S., Compton R., Davies S.G., Evans J. (2001). Biomineralization: Principles and Concepts in Bioinorganic Materials Chemistry.

[2] Meldrum F.C., Cölfen H., Cölfen H. (2008). Controlling Mineral Morphologies and Structures in Biological and Synthetic Systems. Chem. Rev..

[3] Espinosa H.D., Rim J.E., Barthelat F.F., Buehler M.J. (2009). Merger of Structure and Material in Nacre and Bone—Perspectives on de Novo Biomimetic Materials. Prog. Mater. Sci..

[4] Barthelat F., Rabiei R. (2011). Toughness Amplification in Natural Composites. J. Mech. Phys. Solids.

[5] Wegst U.G.K., Bai H., Saiz E., Tomsia A.P., Ritchie R.O. (2014). Bioinspired Structural Materials. Nat. Mater..

[6] Marin F., Luquet G., Marie B., Medakovic D. (2008). Molluscan Shell Proteins: Primary Structure, Origin, and Evolution. Curr. Top. Dev. Biol..

[7] Wilbur K.M., Saleuddin A.S.M. (1983). Shell Formation. The Mollusca, Volume 4.

[8] Marie B., Joubert C., Tayalé A., Zanella-Cléon I., Belliard C., Piquemal D., Cochennec-Laureau N., Marin F., Gueguen Y., Montagnani C. (2012). Different Secretory Repertoires Control the Biomineralization Processes of Prism and Nacre Deposition of the Pearl Oyster Shell. Proc. Natl. Acad. Sci. USA.

[9] Mourea G., Vilarinho L., Santos A.C., Machado J. (2000). Organic Compounds in the Extrapalial Fluid and Haemolymph of Anodonta Cygnea (L.) with Emphasis on the Seasonal Biomineralization Process. Comp. Biochem. Physiol. Part B Biochem. Mol. Biol..

[10] Marin F., Narayanappa P., Motreuil S., Müller W.E.G. (2011). Acidic Shell Proteins of the Mediterranean Fan Mussel *Pinna nobilis*. Molecular Biomineralization. Progress in Molecular and Subcellular Biology.

[11] Thula T.T., Svedlund F., Rodriguez D.E., Podschun J., Pendi L., Gower L.B. (2011). Mimicking the Nanostructure of Bone: Comparison of Polymeric Process-Directing Agents. Polymers.

[12] Marin F., Amons R., Guichard N., Stigter M., Hecker A., Luquet G., Layrolle P., Alcaraz G., Riondet C., Westbroek P. (2005). Caspartin and Calprismin, Two Proteins of the Shell Calcitic Prisms of the Mediterranean Fan Mussel *Pinna nobilis*. J. Biol. Chem..

[13] Marin F., Luquet G., Bäuerlein E. (2007). Unusually Acidic Proteins in Biomineralization. Handbook of Biomineralization: Biological Aspects and Structure Formation.

[14] Hovden R., Wolf S.E., Holtz M.E., Marin F., Muller D.A., Estroff L.A. (2015). Nanoscale Assembly Processes Revealed in the Nacroprismatic Transition Zone of *Pinna nobilis* Mollusc Shells. Nat. Commun..

[15] Teng H.H., Dove P.M., De Yoreo J.J. (2000). Kinetics of Calcite Growth: Surface Processes and Relationships to Macroscopic Rate Laws. Geochim. Cosmochim. Acta.

[16] Nielsen A.E. (1987). Rate Laws and Rate Constants in Crystal Growth. Croat. Chim. Acta.

[17] Vielzeuf D., Garrabou J., Baronnet A., Grauby O., Marschal C. (2008). Nano to Macroscale Biomineral Architecture of Red Coral (Corallium Rubrum). Am. Mineral..

[18] Addadi L., Joester D., Nudelman F., Weiner S. (2006). Mollusk Shell Formation: A Source of New Concepts for Understanding Biomineralization Processes. Chem. Eur. J..

[19] Wolf S.E., Böhm C.F., Harris J., Demmert B., Jacob D.E., Mondeshki M., Ruiz-Agudo E.E., Rodriguez-Navarro C., Rodríguez-Navarro C. (2016). Nonclassical Crystallization in vivo et in vitro (I): Process-Structure-Property Relationships of Nanogranular Biominerals. J. Struct. Biol..

[20] Weiner S., Addadi L. (2011). Crystallization Pathways in Biomineralization. Annu. Rev. Mater. Res..

[21] Watabe N., Wilbur K.M., Saleuuddin A. (1983). Shell Repair. The Mollusca, Physiology, Volume 4, Part 1.

[22] Gower L.B. (2008). Biomimetic Model Systems for Investigating the Amorphous Precursor Pathway and Its Role in Biomineralization. Chem. Rev..

[23] Wolf S.E., Lieberwirth I., Natalio F., Bardeau J.-F., Delorme N., Emmerling F., Barrea R., Kappl M., Marin F. (2012). Merging Models of Biomineralisation with Concepts of Nonclassical Crystallisation: Is a Liquid Amorphous Precursor Involved in the Formation of the Prismatic Layer of the Mediterranean Fan Mussel *Pinna nobilis*?. Faraday Discuss..

[24] Beniash E., Aizenberg J., Addadi L., Weiner S. (1997). Amorphous calcium carbonate transforms into calcite during sea urchin larval spicule growth. Proc. R. Soc. Lond. B Biol. Sci..

[25] Li H., Xin H.L., Kunitake M.E., Keene E.C., Muller D.A., Estroff L.A., Muller A., Estroff L.A. (2011). Calcite Prisms from Mollusk Shells (Atrina Rigida): Swiss-Cheese-like Organic-Inorganic Single-Crystal Composites. Adv. Funct. Mater..

[26] Falini G., Albeck S., Weiner S., Addadi L., Falini G., Albeck S., Weiner S., Addadit L. (1996). Control of Aragonite or Calcite Polymorphism by Mollusk Shell Macromolecules. Science.

[27] Gong Y.U.T., Killian C.E., Olson I.C., Appathurai N.P., Amasino A.L., Martin M.C., Holt L.J., Wilt F.H., Gilbert P.U.P.A. (2012). Phase Transitions in Biogenic Amorphous Calcium Carbonate. Proc. Natl. Acad. Sci. USA.

[28] Radha A.V., Forbes T.Z.T.Z., Killian C.E., Gilbert P.U.P.A., Navrotsky A. (2010). Transformation and Crystallization Energetics of Synthetic and Biogenic Amorphous Calcium Carbonate. Proc. Natl. Acad. Sci. USA.

[29] De Yoreo J.J., Gilbert P.U.P.A., Sommerdijk N.A.J.M., Penn R.L., Whitelam S., Joester D., Zhang H., Rimer J.D., Navrotsky A., Banfield J.F. (2015). Crystallization by Particle Attachment in Synthetic, Biogenic, and Geologic Environments. Science.

[30] Albéric M., Bertinetti L., Zou Z., Fratzl P., Habraken W., Politi Y. (2017). The Crystallization of Amorphous Calcium Carbonate Is Kinetically Governed by Ion Impurities and Water. Adv. Sci..

[31] Killian C.E., Metzler R., Gong Y.U.T., Olson I.C., Aizenberg J., Politi Y., Wilt F.H., Scholl A., Young A., Doran A. (2009). Mechanism of Calcite Co-Orientation in the Sea Urchin Tooth. J. Am. Chem. Soc..

[32] Kim Y.-Y., Schenk A.S., Ihli J., Kulak A.N., Hetherington N.B.J., Tang C.C., Schmahl W.W., Griesshaber E., Hyett G., Meldrum F.C. (2014). A Critical Analysis of Calcium Carbonate Mesocrystals. Nat. Commun..

[33] Gal A., Kahil K., Vidavsky N., DeVol R.T., Gilbert P.U.P.A., Fratzl P., Weiner S., Addadi L. (2014). Particle Accretion Mechanism Underlies Biological Crystal Growth from an Amorphous Precursor Phase. Adv. Funct. Mater..

[34] Gal A., Weiner S., Addadi L. (2015). A Perspective on Underlying Crystal Growth Mechanisms in Biomineralization: Solution Mediated Growth versus Nanosphere Particle Accretion. CrystEngComm.

[35] Harris J., Mey I., Hajir M., Mondeshki M., Wolf S.E. (2015). Pseudomorphic Transformation of Amorphous Calcium Carbonate Films Follows Spherulitic Growth Mechanisms and Can Give Rise to Crystal Lattice Tilting. CrystEngComm.

[36] Gower L.B., Odom D. (2000). Deposition of Calcium Carbonate Films by a Polymer-Induced Liquid-Precursor (PILP) Process. J. Cryst. Growth.

[37] Politi Y., Metzler R.A., Abrecht M., Gilbert B., Wilt F.H., Sagi I., Addadi L., Weinfurter H., Gilbert P.U.P.A. (2008). Transformation Mechanism of Amorphous Calcium Carbonate into Calcite in the Sea Urchin Larval Spicule. Proc. Natl. Acad. Sci. USA.

[38] Gilbert P.U.P.A., Metzler R.A., Zhou D., Scholl A., Doran A., Young A., Kunz M., Tamura N., Coppersmith S.N. (2008). Gradual Ordering in Red Abalone Nacre. J. Am. Chem. Soc..

[39] Hu Q., Nielsen M.H., Freeman C.L., Hamm L.M., Tao J., Lee J.R.I., Han T.Y.J., Becker U., Harding J.H., Dove P.M. (2012). The Thermodynamics of Calcite Nucleation at Organic Interfaces: Classical vs. Non-Classical Pathways. Faraday Discuss..

[40] Cölfen H., Mann S. (2003). Higher-Order Organization by Mesoscale Self-Assembly and Transformation of Hybrid Nanostructures. Angew. Chem. Int. Ed..

[41] Bergström L., Sturm Née Rosseeva E.V., Salazar-Alvarez G., Cölfen H. (2015). Mesocrystals in Biominerals and Colloidal Arrays. Acc. Chem. Res..

[42] Cölfen H. (2006). Bio-Inspired Mineralization Using Hydrophilic Polymers. Top. Curr. Chem..

[43] Robb D.T., Privman V. (2008). Model of Nanocrystal Formation in Solution by Burst Nucleation and Diffusional Growth. Langmuir.

[44] Gower L.B., Tirrell D. (1998). Calcium Carbonate Films and Helices Grown in Solutions of Poly(Aspartate). J. Cryst. Growth.

[45] Rodríguez-Navarroa C., Ruiz-Agudo E., Harris J., Wolf S.E. (2016). Nonclassical crystallization in vivo et in vitro (II): Nanogranular features in biomimetic minerals disclose a general colloid-mediated crystal growth mechanism. J. Struct. Biol..

[46] Wolf S.E., Leiterer J., Kappl M., Emmerling F., Tremel W. (2008). Early Homogenous Amorphous Precursor Stages of Calcium Carbonate and Subsequent Crystal Growth in Levitated Droplets. J. Am. Chem. Soc..

[47] Tartaj P., Amarilla J.M. (2011). Multifunctional Response of Anatase Nanostructures Based on 25 Nm Mesocrystal-Like Porous Assemblies. Adv. Mater..

[48] Zhang A.-Y., Long L.-L., Li W.-W., Wang W.-K., Yu H.-Q. (2013). Hexagonal Microrods of Anatase Tetragonal TiO_2_: Self-Directed Growth and Superior Photocatalytic Performance. Chem. Commun..

[49] Song R.Q., Krasia-Christoforou T., Debus C., Cölfen H. (2017). Structure and Magnetic Property Control of Copper Hydroxide Acetate by Non-Classical Crystallization. Small.

[50] Song R.-Q., Cölfen H. (2010). Mesocrystals-Ordered Nanoparticle Superstructures. Adv. Mater..

[51] Song R.-Q., Cölfen H., Xu A.-W., Hartmann J., Antonietti M. (2009). Polyelectrolyte-Directed Nanoparticle Aggregation: Systematic Morphogenesis of Calcium Carbonate by Nonclassical Crystallization. ACS Nano.

[52] Wang D., Xie T., Peng Q., Li Y. (2008). Ag, Ag2S, and Ag2Se Nanocrystals: Synthesis, Assembly, and Construction of Mesoporous Structures. J. Am. Chem. Soc..

[53] Wang T., Antonietti M., Cölfen H. (2006). Calcite Mesocrystals: “Morphing” Crystals by a Polyelectrolyte. Chemistry.

[54] Wang T., Cölfen H., Antonietti M. (2005). Nonclassical Crystallization: Mesocrystals and Morphology Change of CaCO_3_ Crystals in the Presence of a Polyelectrolyte Additive. J. Am. Chem. Soc..

[55] Yang Y., Yang Y., Wu H., Guo S. (2013). Control of the Formation of Rod-like ZnO Mesocrystals and Their Photocatalytic Properties. CrystEngComm.

[56] Da Silva R.O., Gonçalves R.H., Stroppa D.G., Ramirez A.J., Leite E.R. (2011). Synthesis of Recrystallized Anatase TiO_2_ Mesocrystals with Wulff Shape Assisted by Oriented Attachment. Nanoscale.

[57] Schenk A.S., Eiben S., Goll M., Reith L., Kulak A.N., Meldrum F.C., Jeske H., Wege C., Ludwigs S. (2017). Virus-Directed Formation of Electrocatalytically Active Nanoparticle-Based Co3O4tubes. Nanoscale.

[58] Ross M.B., Ku J.C., Vaccarezza V.M., Schatz G.C., Mirkin C.A. (2015). Nanoscale Form Dictates Mesoscale Function in Plasmonic DNA–Nanoparticle Superlattices. Nat. Nanotechnol..

[59] Ortega S., Ibáñez M., Liu Y., Zhang Y., Kovalenko M.V., Cadavid D., Cabot A. (2017). Bottom-up Engineering of Thermoelectric Nanomaterials and Devices from Solution-Processed Nanoparticle Building Blocks. Chem. Soc. Rev..

[60] Sang L., Zhao Y., Burda C. (2014). TiO2 Nanoparticles as Functional Building Blocks. Chem. Rev..

[61] Wang Y.-W., Christenson H.K., Meldrum F.C. (2013). Confinement Leads to Control over Calcium Sulfate Polymorph. Adv. Funct. Mater..

[62] Uebe R., Schüler D. (2016). Magnetosome Biogenesis in Magnetotactic Bacteria. Nat. Rev. Microbiol..

[63] Smeets P.J.M., Finney A.R., Habraken W.J.E.M., Nudelman F., Friedrich H. (2017). A Classical View on Nonclassical Nucleation. Proc. Natl. Acad. Sci. USA.

[64] Wolf S.E., Gower L.B., van Driessche A.E.S., Kellermeier M., Benning L.G., Gebauer D. (2017). Challenges and Perspectives of the Polymer-Induced Liquid-Precursor Process: The Pathway from Liquid-Condensed Mineral Precursors to Mesocrystalline Products. New Perspectives on Mineral Nucleation and Growth.

[65] Cölfen H., Antonietti M., Cölfen H., Antonietti M. (2008). Mesocrystals and Nonclassical Crystallization.

[66] Niederberger M., Cölfen H. (2006). Oriented Attachment and Mesocrystals: Non-Classical Crystallization Mechanisms Based on Nanoparticle Assembly. Phys. Chem. Chem. Phys..

[67] Wallace A.F., Hedges L.O., Fernandez-Martinez A., Raiteri P., Gale J.D., Waychunas G.A., Whitelam S., Banfield J.F., De Yoreo J.J. (2013). Microscopic Evidence for Liquid-Liquid Separation in Supersaturated CaCO_3_ Solutions. Science.

[68] Kim Y.-Y., Schenk A.S., Walsh D., Kulak A.N., Cespedes O., Meldrum F.C. (2014). Bio-Inspired Formation of Functional Calcite/Metal Oxide Nanoparticle Composites. Nanoscale.

[69] Schenk A.S., Zlotnikov I., Pokroy B., Gierlinger N., Masic A., Zaslansky P., Fitch A.N., Paris O., Metzger T.H., Cölfen H. (2012). Hierarchical Calcite Crystals with Occlusions of a Simple Polyelectrolyte Mimic Complex Biomineral Structures. Adv. Funct. Mater..

[70] Barthelat F., Rim J.E., Espinosa H.D., Bhushan B., Fuchs H. (2009). A Review on the Structure and Mechanical Properties of Mollusk Shells—Perspectives on Synthetic Biomimetic Materials. Applied Scanning Probe Methods XIII, Biomimetics and Industrial Applications.

[71] Villanova J., Kozachkevich S., Zaslansky P., Kundanati L., Bracha A.A., Polishchuk I., Bloch L., Levy D., Katsman A., Giacobbe C. (2017). Coherently Aligned Nanoparticles within a Biogenic Single Crystal: A Biological Prestressing Strategy. Science.

[72] Sethmann I., Hinrichs R., Wörheide G., Putnis A. (2006). Nano-Cluster Composite Structure of Calcitic Sponge Spicules—A Case Study of Basic Characteristics of Biominerals. J. Inorg. Biochem..

[73] Addadi L., Weiner S. (2014). Biomineralization: Mineral Formation by Organisms. Phys. Scr..

[74] Weiner S., Addadi L. (1997). Design Strategies in Mineralized Biological Materials. J. Mater. Chem..

[75] Gebauer D., Wolf S.E. (2019). Designing Solid Materials from their Solute State: a Shift in Paradigms toward a Holistic Approach in Functional Materials Chemistry. J. Am. Chem. Soc..

[76] Wegst U.G.K., Ashby M.F. (2004). The Mechanical Efficiency of Natural Materials. Philos. Mag..

[77] Meyers M.A., Chen P.-Y., Lin A.Y.-M., Seki Y. (2008). Biological Materials: Structure and Mechanical Properties. Prog. Mater. Sci..

[78] Pokroy B., Zolotoyabko E. (2003). Microstructure of Natural Plywood-like Ceramics: A Study by High-Resolution Electron Microscopy and Energy-Variable X-Ray Diffraction. J. Mater. Chem..

[79] Tai K., Ulm F.J., Ortiz C. (2006). Nanogranular Origins of the Strength of Bone. Nano Lett..

[80] Ryall R.L., Fleming D.E., Doyle I.R., Evans N.A., Dean C.J., Marshall V.R. (2001). Intracrystalline Proteins and the Hidden Ultrastructure of Calcium Oxalate Urinary Crystals: Implications for Kidney Stone Formation. J. Struct. Biol..

[81] Böhm C.F., Demmert B., Harris J., Fey T., Marin F., Wolf S.E. (2016). Structural Commonalities and Deviations in the Hierarchical Organization of Crossed-Lamellar Shells: A Case Study on the Shell of the Bivalve Glycymeris Glycymeris. J. Mater. Res..

[82] Schenk A.S., Kim Y.Y. (2015). Unraveling the Internal Microstructure of Biogenic and Bioinspired Calcite Single Crystals. MRS Bull..

[83] Mutvei H., Dunca E. (2010). Crystalline Structure, Orientation and Nucleation of the Nacreous Tablets in the Cephalopod Nautilus. Paläontologische Zeitschrift.

[84] Checa A.G., Mutvei H., Osuna-Mascaró A.J., Bonarski J.T., Faryna M., Berent K., Pina C.M., Rousseau M., Macías-Sánchez E. (2013). Crystallographic Control on the Substructure of Nacre Tablets. J. Struct. Biol..

[85] Athanasiadou D., Jiang W., Goldbaum D., Saleem A., Basu K., Pacella M.S., Böhm C.F., Chromik R.R., Hincke M.T., Rodríguez-Navarro A.B. (2018). Nanostructure, Osteopontin, and Mechanical Properties of Calcitic Avian Eggshell. Sci. Adv..

[86] Rodríguez-Navarro A.B., Marie P., Nys Y., Hincke M.T., Gautron J. (2015). Amorphous Calcium Carbonate Controls Avian Eggshell Mineralization: A New Paradigm for Understanding Rapid Eggshell Calcification. J. Struct. Biol..

[87] Seto J., Ma Y., Davis S.A., Meldrum F.C., Gourrier A., Kim Y.-Y., Schilde U., Sztucki M., Burghammer M., Maltsev S. (2012). Structure-Property Relationships of a Biological Mesocrystal in the Adult Sea Urchin Spines. Proc. Natl. Acad. Sci. USA.

[88] Dauphin Y., Cuif J.P., Doucet J., Salomé M., Susini J., Willams C.T. (2003). In Situ Chemical Speciation of Sulfur in Calcitic Biominerals and the Simple Prism Concept. J. Struct. Biol..

[89] Dauphin Y. (2008). The Nanostructural Unity of Mollusc Shells. Mineral. Mag..

[90] Jacob D.E., Soldati A., Wirth R., Huth J., Wehrmeister U., Hofmeister W. (2008). Nanostructure, Composition and Mechanisms of Bivalve Shell Growth. Geochim. Cosmochim. Acta.

[91] Checa A.G., Bonarski J.T., Willinger M.G., Faryna M., Berent K., Kania B., González-Segura A., Pina C.M., Pospiech J., Morawiec A. (2013). Crystallographic Orientation Inhomogeneity and Crystal Splitting in Biogenic Calcite. J. R. Soc. Interface.

[92] Okumura T., Suzuki M., Nagasawa H., Kogure T. (2012). Microstructural Variation of Biogenic Calcite with Intracrystalline Organic Macromolecules. Cryst. Growth Des..

[93] Suzuki M., Kameda J., Sasaki T., Saruwatari K., Nagasawa H., Kogure T. (2010). Characterization of the Multilayered Shell of a Limpet, Lottia Kogamogai (Mollusca: Patellogastropoda), Using SEM–EBSD and FIB–TEM Techniques. J. Struct. Biol..

[94] Aizenberg J., Hanson J., Koetzle T.F., Weinfurter H., Addadi L., York N., August R.V. (1997). Control of Macromolecule Distribution within Synthetic and Biogenic Single Calcite Crystals. J. Am. Chem. Soc..

[95] Berman A., Addadi L., Kvick A.Y.E., Leiserowitz L., Nelson M., Weinfurter H. (1990). Intercalation of Sea Urchin Proteins in Calcite: Study of a Crystalline Composite Material. Science.

[96] Pisklak D.M., Szeleszczuk Ł., Wawer I. (2012). 1H and 13C Magic-Angle Spinning Nuclear Magnetic Resonance Studies of the Chicken Eggshell. J. Agric. Food Chem..

[97] Ben Shir I., Kababya S., Katz I., Pokroy B., Schmidt A. (2013). Exposed and Buried Biomineral Interfaces in the Aragonitic Shell of *Perna canaliculus* Revealed by Solid-State NMR. Chem. Mater..

[98] Lee D., Leroy C., Crevant C., Bonhomme-Coury L., Babonneau F., Laurencin D., Bonhomme C., De Paëpe G., Bryce D.L., Laurencin D. (2017). Interfacial Ca^2+^ Environments in Nanocrystalline Apatites Revealed by Dynamic Nuclear Polarization Enhanced 43Ca NMR Spectroscopy. Nat. Commun..

[99] Agbaje O.B.A., Ben Shir I., Zax D.B., Schmidt A., Jacob D.E. (2018). Biomacromolecules within Bivalve Shells: Is Chitin Abundant?. Acta Biomater..

[100] Bar-On B., Wagner H.D. (2013). Structural Motifs and Elastic Properties of Hierarchical Biological Tissues—A Review. J. Struct. Biol..

[101] Meyers M.A., Lin A., Chen P., Muyco J. (2008). Mechanical Strength of Abalone Nacre: Role of the Soft Organic Layer. J. Mech. Behav. Biomed. Mater..

[102] Liu Z., Meyers M.A., Zhang Z., Ritchie R.O. (2017). Functional Gradients and Heterogeneities in Biological Materials: Design Principles, Functions, and Bioinspired Applications. Prog. Mater. Sci..

[103] Currey J.D., Taylor J.D. (1974). The Mechanical Behaviour of Some Molluscan Hard Tissues. J. Zool..

[104] Chen P.-Y., McKittrick J., Meyers M.A. (2012). Biological Materials: Functional Adaptations and Bioinspired Designs. Prog. Mater. Sci..

[105] Bayerlein B., Bertinetti L., Bar-on B., Blumtritt H., Fratzl P. (2016). Inherent Role of Water in Damage Tolerance of the Prismatic Mineral-Organic Biocomposite in the Shell of *Pinna nobilis*. Adv. Fcunt. Mater..

[106] Cuif J.-P., Burghammer M., Chamard V., Dauphin Y., Godard P., Moullac G., Nehrke G., Perez-Huerta A. (2014). Evidence of a Biological Control over Origin, Growth and End of the Calcite Prisms in the Shells of Pinctada Margaritifera (Pelecypod, Pterioidea). Minerals.

[107] Nakahara H., Kakei M., Bevelander G. (1980). Fine Structure and Amino Acid Composition of the Organic “Envelope” in the Prismatic Layer of Some Bivlave Shells. Jpn. J. Malacol..

[108] Gilbert P.U.P.A., Young A., Coppersmith S.N. (2011). Measurement of C-Axis Angular Orientation in Calcite (CaCO3) Nanocrystals Using X-Ray Absorption Spectroscopy. Proc. Natl. Acad. Sci. USA.

[109] Kunitake M.E., Mangano L.M., Peloquin J.M., Baker S.P., Estroff L.A. (2013). Evaluation of Strengthening Mechanisms in Calcite Single Crystals from Mollusk Shells. Acta Biomater..

[110] Kuhn-Spearing L.T., Kessler H., Chateau E., Ballarini R., Heuer A.H., Spearing S.M. (1996). Fracture Mechanisms of the Strombus Gigas Conch Shell: Implications for the Design of Brittle Laminates. J. Mater. Sci..

[111] Kamat S., Su X., Ballarini R., Heuer A. (2000). Structural Basis for the Fracture Toughness of the Shell of the Conch Strombus Gigas. Nature.

[112] Weaver J.C., Milliron G.W., Miserez A., Evans-Lutterodt K., Herrera S., Gallana I., Mershon W.J., Swanson B., Zavattieri P., DiMasi E. (2012). The Stomatopod Dactyl Club: A Formidable Damage-Tolerant Biological Hammer. Science.

[113] Grunenfelder L.K., Suksangpanya N., Salinas C., Milliron G., Yaraghi N., Herrera S., Evans-Lutterodt K., Nutt S.R., Zavattieri P., Kisailus D. (2014). Bio-Inspired Impact-Resistant Composites. Acta Biomater..

[114] Guarín-Zapata N., Gomez J., Yaraghi N., Kisailus D., Zavattieri P.D. (2015). Shear Wave Filtering in Naturally-Occurring Bouligand Structures. Acta Biomater..

[115] de Obaldia E.E., Jeong C., Grunenfelder L.K., Kisailus D., Zavattieri P. (2015). Analysis of the Mechanical Response of Biomimetic Materials with Highly Oriented Microstructures through 3D Printing, Mechanical Testing and Modeling. J. Mech. Behav. Biomed. Mater..

[116] Mirkhalaf M., Dastjerdi A.K., Barthelat F. (2014). Overcoming the Brittleness of Glass through Bio-Inspiration and Micro-Architecture. Nat. Commun..

[117] Ritchie R.O. (2011). The Conflicts between Strength and Toughness. Nat. Mater..

[118] Weißbach W., Dahms M., Jaroschek C. (2015). Werkstoffkunde.

[119] Turner F.J., Griffs D.T., Heard H. (1954). Experimental Deformation of Calcite Crystals. Geol. Soc. Am. Bull..

[120] Carlton C.E., Ferreira P.J. (2007). What Is behind the Inverse Hall-Petch Effect in Nanocrystalline Materials?. Acta Mater..

[121] Wiederhorn S.M. (1984). Brittle Fracture and Toughening Mechanisms in Ceramics. Annu. Rev. Mater. Sci..

[122] Salmang H., Scholze H. (2007). Keramik.

[123] Schamel M., Barralet J.E., Gelinsky M., Groll J., Gbureck U. (2017). Intrinsic 3D Prestressing: A New Route for Increasing Strength and Improving Toughness of Hybrid Inorganic Biocements. Adv. Mater..

[124] Barthelat F., Tang H., Zavattieri P., Li C., Espinosa H. (2007). On the Mechanics of Mother-of-Pearl: A Key Feature in the Material Hierarchical Structure. J. Mech. Phys. Solids.

[125] Fantner G.E., Hassenkam T., Kindt J.H., Weaver J.C., Birkedal H., Pechenik L., Cutroni J.A., Cidade G.A.G., Stucky G.D., Morse D.E. (2005). Sacrificial Bonds and Hidden Length Dissipate Energy as Mineralized Fibrils Separate during Bone Fracture. Nat. Mater..

[126] Li X., Xu Z.-H., Wang R. (2006). In Situ Observation of Nanograin Rotation and Deformation in Nacre. Nano Lett..

[127] Gordon L.M., Joester D. (2011). Nanoscale Chemical Tomography of Buried Organic-Inorganic Interfaces in the Chiton Tooth. Nature.

[128] Barthelat F.F. (2007). Biomimetics for next Generation Materials. Philos. Trans. A Math. Phys. Eng. Sci..

[129] Taylor J.D., Layman M. (1972). The Mechanical Properties of Bivalve (Mollusca) Shell Structure. Paleontology.

[130] Marin F., Luquet G. (2004). Molluscan Shell Proteins. C. R. Palevol.

[131] Checa A.G., Cartwright J.H.E., Willinger M.G. (2011). Mineral Bridges in Nacre. J. Struct. Biol..

[132] Wang R.Z., Suo Z., Evans A.G., Yao N., Aksay I.A. (2011). Deformation Mechanisms in Nacre. J. Mater. Res..

[133] Heinemann F., Launspach M., Gries K., Fritz M. (2011). Gastropod Nacre: Structure, Properties and Growth—Biological, Chemical and Physical Basics. Biophys. Chem..

[134] Barthelat F., Li C.-M., Comi C., Espinosa H.D. (2006). Mechanical Properties of Nacre Constituents and Their Impact on Mechanical Performance. J. Mater. Res..

[135] Weber E., Pokroy B. (2015). Intracrystalline Inclusions within Single Crystalline Hosts: From Biomineralization to Bio-Inspired Crystal Growth. CrystEngComm.

[136] Levi-Kalisman Y., Falini G., Addadi L., Weinfurter H. (2001). Structure of the Nacreous Organic Matrix of a Bivalve Mollusk Shell Examined in the Hydrated State Using Cryo-TEM. J. Struct. Biol..

[137] Younis S., Kauffmann Y., Bloch L., Zolotoyabko E. (2012). Inhomogeneity of Nacre Lamellae on the Nanometer Length Scale. Cryst. Growth Des..

[138] Kim Y.-Y., Carloni J.D., Demarchi B., Sparks D., Reid D.G., Kunitake M.E., Tang C.C., Duer M.J., Freeman C.L., Pokroy B. (2016). Tuning Hardness in Calcite by Incorporation of Amino Acids. Nat. Mater..

[139] Koyama M., Zhang Z., Wang M., Ponge D., Raabe D., Tsuzaki K., Noguchi H., Tasan C.C. (2017). Bone-like Crack Resistance in Hierarchical Metastable Nanolaminate Steels. Science.

[140] Cao S.C., Liu J., Zhu L., Li L., Dao M., Lu J., Ritchie R.O. (2018). Nature-Inspired Hierarchical Steels. Sci. Rep..

[141] Wat A., Lee J.I., Ryu C.W., Gludovatz B., Kim J., Tomsia A.P., Ishikawa T., Schmitz J., Meyer A., Alfreider M. (2019). Bioinspired Nacre-like Alumina with a Bulk-Metallic Glass-Forming Alloy as a Compliant Phase. Nat. Commun..

[142] Kilper S., Facey S.J., Burghard Z., Hauer B., Rothenstein D., Bill J. (2018). Macroscopic Properties of Biomimetic Ceramics Are Governed by the Molecular Recognition at the Bioorganic—Inorganic Interface. Adv. Funct. Mater..

[143] Eder M., Amini S., Fratzl P. (2018). Biological Composites—Complex Structures for Functional Diversity. Science.

[144] Aizenberg J., Weiner S., Tkachenko A., Addadi L., Hendler G. (2001). Calcitic Microlenses as Part of the Photoreceptor System in Brittlestars. Nature.

[145] Nys Y., Gautron J., Anton M., Schade R., Huopalahti R., López-Fandino R. (2007). Structure and Formation of the Eggshell. Bioactive Egg Compounds.

[146] Aizenberg J., Weaver J.C., Thanawala M.S., Sundar V.C., Morse D.E., Fratzl P. (2005). Skeleton of *Euplectella* sp.: Structural Hierarchy from the Nanoscale to the Macroscale. Science.

[147] Weaver J.C., Aizenberg J., Fantner G.E., Kisailus D., Woesz A., Allen P., Fields K., Porter M.J., Zok F.W., Hansma P.K. (2007). Hierarchical Assembly of the Siliceous Skeletal Lattice of the Hexactinellid Sponge Euplectella Aspergillum. J. Struct. Biol..

[148] Ehrlich H., Deutzmann R., Brunner E., Cappellini E., Koon H., Solazzo C., Yang Y., Ashford D., Thomas-Oates J., Lubeck M. (2010). Mineralization of the Metre-Long Biosilica Structures of Glass Sponges Is Templated on Hydroxylated Collagen. Nat. Chem..

[149] Ehrlich H. (2010). Chitin and Collagen as Universal and Alternative Templates in Biomineralization. Int. Geol. Rev..

[150] Kröger N., Deutzmann R., Sumper M. (1999). Polycationic Peptides from Diatom Biosilica That Direct Silica Nanosphere Formation. Science.

[151] Matsunaga S., Sakai R., Jimbo M., Kamiya H. (2007). Long-Chain Polyamines (LCPAs) from Marine Sponge: Possible Implication in Spicule Formation. Chembiochem.

[152] Poulsen N., Kröger N. (2004). Silica Morphogenesis by Alternative Processing of Silaffins in the Diatom Thalassiosira Pseudonana. J. Chem. Phys..

[153] Shimizu K., Cha J., Stucky G.D. (1998). Silicatein: Cathepsin L-like Protein in Sponge Biosilica. Proc. Natl. Acad. Sci. USA.

[154] Sundar V.C., Yablon A.D., Grazul J.L., Ilan M., Aizenberg J. (2003). Fibre-Optical Features of a Glass Sponge. Nature.

[155] Müller W.E.G., Wolf S.E., Schlossmacher U., Wang X.-H., Boreiko A., Brandt D., Tremel W., Schröder H.-C. (2008). Poly(Silicate)-Metabolizing Silicatein in Siliceous Spicules and Silicasomes of Demosponges Comprises Dual Enzymatic Activities (Silica Polymerase and Silica Esterase). FEBS J..

[156] Cha J.N., Shimizu K., Zhou Y., Christiansen S.C., Chmelka B.F., Stucky G.D., Morse D.E. (1999). Silicatein Filaments and Subunits from a Marine Sponge Direct the Polymerization of Silica and Silicones in Vitro. Proc. Natl. Acad. Sci. USA.

[157] Zhou Y., Shimizu K., Cha J.N., Stucky G.D., Morse D.E. (1999). Efficient Catalysis of Polysiloxane Synthesis by Silicatein α Requires Specific Hydroxy and Imidazole Functionalities. Angew. Chem. Int. Ed..

[158] Shimizu K., Morse D.E. (2018). Silicatein: A Unique Silica-Synthesizing Catalytic Triad Hydrolase From Marine Sponge Skeletons and Its Multiple Applications. Methods Enzymol..

[159] Brutchey R.L., Morse D.E. (2008). Silicatein and the Translation of Its Molecular Mechanism of Biosilicification into Low Temperature Nanomaterial Synthesis. Chem. Rev..

[160] Kisailus D., Choi J.H., Weaver J.C., Yang W., Morse D.E. (2005). Enzymatic Synthesis and Nanostructural Control of Gallium Oxide at Low Temperature. Adv. Mater..

[161] Sumerel J.L., Yang W.J., Kisailus D., Weaver J.C., Choi J.H., Morse D.E. (2003). Biocatalytically Templated Synthesis of Titanium Dioxide. Chem. Mater..

[162] Curnow P., Bessette P.H., Kisailus D., Murr M.M., Daugherty P.S., Morse D.E. (2005). Enzymatic Synthesis of Layered Titanium Phosphates at Low Temperature and Neutral PH by Cell-Surface Display of Silicatein-α. J. Am. Chem. Soc..

[163] Wolf S.E., Schlossmacher U., Pietuch A., Mathiasch B., Schröder H.-C., Müller W.E.G., Tremel W. (2010). Formation of Silicones Mediated by the Sponge Enzyme Silicatein-α. Dalton Trans..

[164] O’Leary P., van Walree C.A., Mehendale N.C., Sumerel J., Morse D.E., Kaska W.C., van Koten G., Gebbink R.J.M. (2009). Enzymatic Immobilization of Organometallic Species: Biosilification of NCN- and PCP-Pincer Metal Species Using Demosponge Axial Filaments. Dalton Trans..

[165] Tahir M.N., Théato P., Müller W.E.G., Schröder H.-C., Janshoff A., Zhang J., Huth J., Tremel W. (2004). Monitoring the Formation of Biosilica Catalysed by Histidine-Tagged Silicatein. Chem. Commun..

[166] Tahir M.N., Théato P., Müller W.E.G., Schröder H.C., Borejko A., Faiß S., Janshoff A., Huth J., Tremel W. (2005). Formation of Layered Titania and Zirconia Catalysed by Surface-Bound Silicatein. Chem. Commun..

[167] Tahir M.N., Eberhardt M., Therese H.A., Kolb U., Theato P., Müller W.E.G., Schröder H.-C., Tremel W. (2006). From Single Molecules to Nanoscopically Structured Functional Materials: Au Nanocrystal Growth on TiO_2_ Nanowires Controlled by Surface-Bound Silicatein. Angew. Chem. Int. Ed..

[168] Shukoor M.I., Natalio F., Therese H.A., Tahir M.N., Ksenofontov V., Panthöfer M., Eberhardt M., Theato P., Schröder H.C., Müller W.E.G. (2008). Fabrication of a Silica Coating on Magnetic γ-Fe_2_O_3_ Nanoparticles by an Immobilized Enzyme. Chem. Mater..

[169] Natalio F., Link T., Müller W.E.G., Schröder H.C., Cui F.-Z., Wang X., Wiens M. (2010). Bioengineering of the Silica-Polymerizing Enzyme Silicatein-α for a Targeted Application to Hydroxyapatite. Acta Biomater..

[170] Natalio F., Corrales T.P., Panthofer M., Schollmeyer D., Lieberwirth I., Muller W.E.G., Kappl M., Butt H.-J., Tremel W. (2013). Flexible Minerals: Self-Assembled Calcite Spicules with Extreme Bending Strength. Science.

[171] Cha J.N., Stucky G.D., Morse D.E., Deming T.J. (2000). Biomimetic Synthesis of Ordered Silica Structures Mediated by Block Copolypeptides. Nature.

[172] Adamson D.H., Dabbs D.M., Pacheco C.R., Giotto M.V., Morse D.E., Aksay I.A. (2007). Non-Peptide Polymeric Silicatein α Mimic for Neutral PH Catalysis in the Formation of Silica. Macromolecules.

[173] Robinson D.B., Rognlien J.L., Bauer C.A., Simmons B.A. (2007). Dependence of Amine-Accelerated Silicate Condensation on Amine Structure. J. Mater. Chem..

[174] Kisailus D., Najarian M., Weaver J.C., Morse D.E. (2005). Functionalized Gold Nanoparticles Mimic Catalytic Activity of a Polysiloxane-Synthesizing Enzyme. Adv. Mater..

[175] Roth K.M., Zhou Y., Yang W., Morse D.E. (2005). Bifunctional Small Molecules Are Biomimetic Catalysts for Silica Synthesis at Neutral PH. J. Am. Chem. Soc..

[176] Luckarift H.R., Spain J.C., Naik R.R., Stone M.O. (2004). Enzyme Immobilization in a Biomimetic Silica Support. Nat. Biotechnol..

[177] Corma A., Díaz-Cabañas M.J., Moliner M., Rodríguez G. (2006). Synthesis of Micro- and Mesoporous Molecular Sieves at Room Temperature and Neutral PH Catalyzed by Functional Analogues of Silicatein. Chem. Commun..

